# Specialist and Primary Physicians’ Experiences and Perspectives of Collaboration While Caring for Palliative Patients—A Qualitative Study

**DOI:** 10.3390/healthcare11152188

**Published:** 2023-08-02

**Authors:** Siri Andreassen Devik, Gunhild Lein Lersveen

**Affiliations:** 1 Centre for Care Research, Mid-Norway, Faculty of Nursing and Health Sciences, Nord University, 8026 Bodø, Norway; 2 Centre for Development of Institutional and Home Care Services in Trøndelag, 7650 Verdal, Norway; gunhild.lersveen@afjord.kommune.no

**Keywords:** palliative care, collaboration, physicians, home, hospital, pair interviewing, qualitative research

## Abstract

Increasing numbers of people living with chronic and life-limiting diseases are actualising a greater need for palliative care. Physicians are an important provider for identifying the need for palliation, and effective follow-up requires physician collaboration across different service levels. This study aimed to explore and describe how physicians in hospitals and municipalities experience their roles and interactions in the care of palliative patients. Pair interviews were performed with seven physicians working in hospitals, primary care and nursing homes in Mid-Norway. Systematic text condensation was used to analyse the data, resulting in three main themes: *The boundaries of palliative care*, *Alternating understandings of roles* and *Absence of planning*. The physicians’ interactions with palliative patients appeared as a fragmented distribution of tasks rather than a real collaboration with shared responsibility. At both levels, the physicians seemed to assume withdrawn roles as a reaction to unclear and unspoken expectations and to avoid interfering with others’ responsibilities. Moreover, their understanding of palliative care and which groups should be included varied. Realising a collaboration between physicians that is beneficial for both patients and physicians, greater openness and real arenas for discussion and decision-making support are required.

## 1. Introduction

Changes in the global population’s age composition and lifestyle mean more people are living longer with serious illnesses and palliative needs [1,2]. Reduction in hospital stays, more outpatient treatment and patients’ preferences also place a great responsibility on primary healthcare services [1,3]. Accordingly, a collaboration between professionals at various levels is crucial for ensuring high-quality palliative treatment and care and patients’ experiences [4].

In Norway, palliative care adheres to the guidelines established by the European Association for Palliative Care (EAPC) [5]. It includes a holistic approach that ensures that people with life-limiting conditions have access to interdisciplinary palliative treatment, which can be initiated early in the course of the illness [6]. The Norwegian health authorities have pointed to the need for more interdisciplinary collaboration in the practice of palliative care and have promoted incentives to increase interaction between general practitioners (GPs) and specialists in hospitals [7]. By becoming involved in the palliative care process, GPs can provide effective treatment for less serious ailments, determine priority admissions and obtain expertise from the specialist health service if necessary [8]. While the primary health service is expected to increasingly take responsibility for patients in need of palliative care, the degree of collaboration between professionals varies from well-organised teamwork to fragmented services in Norway [9] as seen in other European countries [4]. Both GPs and other health personnel in Norwegian municipalities have reported that a lack of communication both within their level and between specialists and the primary healthcare service is a key factor in worst-case patient scenarios [10].

Access to and provision of services are negatively affected by a lack of integration and collaboration in palliative care. For example, patients’ preferences regarding the place of death are only met in a small number of cases [8,11]. One Norwegian study [12] showed that only a minority of patients receive home visits from their GP, even though the service is seen as a prerequisite for a dignified end of life at home. GPs in Norway have reported that they are rarely involved in death at home, which limits their experience and opportunity for competence development [13]. GPs have also stated little consensus exists concerning their role in palliative care and, consequently, their degree of involvement covers the whole spectrum from high involvement to not being involved at all [14]. Fasting et al. [14] described that although GPs felt that they had the competence to provide basic symptom relief and psychosocial as well as existential care for their palliative patients, they were also dependent on support from the specialist health service. However, the GPs expressed that the specialist health service mainly concentrates on cancer patients and that specialist support for multimorbid patients is more difficult to obtain. 

Medical collaboration in palliative care may encounter several barriers. GPs in the Netherlands [15] reported specific challenges when patients continue to receive hospital treatment and when patients have non-oncological conditions where the transition from curative to palliative care is not as clearly defined. The specialists tended to delay referring to palliative care, and the GPs experienced the communication as insufficient and unclear [15]. Despite strong recommendations to initiate palliative care early in the course of the disease [16], referrals happen too late and often not before death is imminent [17,18]. Part of the challenge may relate to difficulties in identifying which patients are eligible for palliative care, especially because chronic progressive diseases often follow less predictable disease trajectories [1,19,20]. Diagnoses associated with death, especially cancer diagnoses, are significantly more likely to receive palliative care compared to patients with organ failure and ‘frail’ patients [21].

According to Flierman et al. [22], efforts should be made to increase knowledge and skills around the identification of palliative needs and communication with patients about the end of life, especially in the hospital setting. A systematic review [23] revealed that oncologists and haematologists viewed referral to palliative care as abandonment, a disruption in the therapeutic relationship, and a loss of hope. Furthermore, the hospital environment poses various obstacles, including physical and organisational challenges, which have been demonstrated to impact physicians’ communication with patients [24]. Additionally, the communication among physicians across different care settings involved in the patient’s palliative care has rarely been investigated [25]. Our own review of the literature supports this, as it seems to be characterised by interdisciplinary perspectives and a limited number of studies that exclusively focus on the physician’s perspective on collaboration.

This study, therefore, intended to bring together specialist and primary care physician perspectives for joint discussions. While interaction and integration in the health service have mainly been studied and explained from a structural perspective, this study also considers the social and relational aspects of interaction [26,27]. Overall, this study aimed to explore and describe how physicians in hospitals and municipalities experience their roles and interactions in the care of palliative patients.

## 2. Materials and Methods

### 2.1. Design

To gain insight into different physicians’ experiences of their roles and interactions in the care of palliative patients across service levels, we used paired interviewing [28,29] with physicians from both hospitals and municipalities. This method is suitable for collecting data about how the pair perceives the same phenomenon [30].

### 2.2. Setting

The study took place in a Norwegian region where two local hospitals are responsible for providing specialist healthcare services to approximately 140,000 inhabitants in 19 municipalities. One hospital has 179 somatic beds (hospital beds that are designated for patients requiring medical or surgical treatment for physical or non-psychiatric conditions), while the other has 91 beds. Both hospitals have interdisciplinary palliative care teams that assist municipalities with the care of palliative patients in nursing homes and home care services. The municipalities covered by the hospital services vary from small rural municipalities with 600 inhabitants to medium-sized urban municipalities with 22,000 inhabitants. As part of the public healthcare system, palliative care in Norway has gradually developed at all levels of care since the 80s, but mainly with cancer patients as the target group [31]. The medical services in the municipalities are organised in a GP model where each resident has a designated GP who is responsible for medical follow-up and referrals to other healthcare services. At the municipal level, each nursing home has a certain number of palliative beds, and the home care services have a coordinator position for palliative care services, usually occupied by a cancer nurse [32]. The nursing homes are also part of the responsibility of the municipal healthcare service in Norway, where patients are followed up by either GPs by appointment or a nursing home physician. Both the GP scheme and medical services in nursing homes fall under the responsibility of the municipality. Therefore, the physicians in this article are referred to as municipal physicians.

Medical education in Norway takes 6 years and leads to a cand.med. degree, which corresponds to a Doctor of Medicine. The programme includes extensive clinical service that covers a wide range of patients, from primary health care in municipalities to centralised specialist hospital departments, and from emergency medicine to caring for chronically ill patients. Cand. med. candidates must complete 1.5 years of practice to obtain legal authorisation to practice medicine in Norway. The practical study is part of specialist education which has a number of different possibilities. In the case of GPs, specialisation in general medicine is required to be able to hold such a position. 

Four of the municipalities in the hospitals’ catchment area conducted a collaborative project in 2018–2019 to increase competence in palliative care of nursing personnel working in municipal care services. The findings of the project indicated a need for more knowledge about the physicians’ perspectives, both from GPs in municipal healthcare services and medical specialists in hospitals.

### 2.3. Recruitment and Participants

The recruitment of physicians involved inquiries to municipal chief physicians, GP offices and nursing homes in four municipalities (two urban and two rural) with populations ranging from 2600 to 15,000. Physicians at hospitals were recruited via practice consultants in medical and surgical wards. The participants were informed of the purpose of the study in writing and orally and gave written consent if they wanted to participate. Inclusion was based on volunteerism and the participant’s assessment of their experience in palliative care. Participants were also promised fees for lost work profits. Since the project took place during the COVID-19 pandemic, online interviews were conducted at a mutually agreeable time. Even with an extended data collection period (June 2020–March 2021) and many inquiries, we experienced challenges with recruitment and eventually ended up with an uneven number (N = 7) of participants. This uneven sample meant we had to conduct one interview with three physicians: one hospital physician and two physicians from a municipal setting.

### 2.4. Data Collection

The data collection was performed by using pair interviewing: One interview with a GP, a hospital physician and a nursing home physician (Group 1) and two interviews with a GP and a physician from a hospital in each group (Groups 2 and 3). The interviews were based on a semi-structured interview guide sent to the participants in advance. The interview guide was developed based on previous research and identified themes from the collaborative project in 2018–2019. The guide consisted of seven questions. Initial questions asked participants to describe their understanding of the concept of palliative care and who they would define as palliative care patients. Subsequent questions asked them about their perceptions of their role as well as others in the treatment and follow-up of palliative care patients, especially what they found to be working well and less well. Participants were also asked about who they considered to be the central partners, how they experienced cooperation with relatives and their thoughts on what promotes and inhibits optimal interaction during the treatment and follow-up of palliative care patients. Participants were encouraged to respond to each other’s answers and discuss differences in their perspectives. All the interviews took place online via Teams. Both authors participated in all the interviews and functioned as the moderator and co-moderator. The audio recordings were subsequently transcribed verbatim.

### 2.5. Analysis

The interview texts were analysed using the method of systematic text condensation [33], which is an iterative four-step process based on elements of Giorgi’s psychological phenomenological analysis [34]. In the first step, the two authors aimed for a general impression of the whole, looking for preliminary themes associated with the physicians’ views on palliative care and their experiences of interactions while caring for these patients. The authors first read the transcripts separately and then came together to discuss similarities and differences in what they perceived as pertinent topics. In the next step, the transcripts were examined line by line to identify units of meaning that could be sorted under the preliminary topics proposed in the first step. In the third step, meaning units were arranged to form sub-themes. In all these steps, the preliminary themes were adjusted. In the fourth step, a narrative condensate was created for each theme and its sub-themes and meaning units, and an analytical text was produced to reflect the findings. Throughout the process, the authors continuously discussed the interpretations and went back and forth between steps to ensure these reflected the physicians’ statements in the interview text.

## 3. Results

Seven physicians, including five women and two men, participated in this study. They were aged 40 to 65 years (median = 53 years) and their work experience varied from 14 to 41 years (median = 28 years). The three hospital physicians, who were all senior physicians, covered specialist expertise in surgery, palliation and internal medicine, while the physicians in the municipal setting had specialisation in general medicine. All the physicians reported having significant experience with palliative patients, and two of the hospital physicians had specific expertise in treating cancer diagnoses. One of them had worked for 10 years in a specialised department for palliative care. When asked about their self-assessment of competence in palliative care, the physicians consistently expressed feeling confident in their professional abilities and indicated that they sought assistance from colleagues or relevant specialists when needed. The interviews lasted between 59 and 90 min.

The analysis of the data resulted in three main themes described with analytical text and quotes from the participants.

### 3.1. Theme 1: The Boundaries of Palliative Care

A consensus emerged among the physicians that palliative treatment is aimed at people with an incurable disease, with a limited lifespan and where relief is central to the treatment:

*They* [palliative patients] *are the ones who are at the end of their lives. Where the curative possibilities are in any case degraded and who are considered so poor that one sees that it is relief that is the main focus.*

(GP, Group 1)

The main focus was on patients in the last stage of life, but to meet the goal of the best possible quality of life, it was pointed out that it might be relevant to think about palliative care in the early stages of an incurable disease:

*But also, in those patients who have a long-life expectancy and have moderate ailments, it is important to try and treat the moderate ailments as well as possible to improve the quality of life. That is the goal of palliative care.*

(Hospital physician, Group 2)

The physicians in the municipal health service described how palliative care had gone from being a speciality linked to cancer to becoming a treatment that encompasses all patient groups:

*I think that the group is getting bigger and bigger…. In the past, cancer patients were the classic, but now…. The palliative care patients are also very much those who do not have a malignant disease.*

(GP2, Group 3)

The participants described that the palliative principles were useful regardless of diagnosis, whether the patient had slowly progressing dementia or advanced cancer. The broad approach among the physicians in the municipal health service related to diagnosis and use of palliative principles contrasted with hospital physicians’ opinions. In particular, the hospital physicians made a clear distinction between a cancer diagnosis and other diagnoses:

*For me, the palliative care patient is a cancer patient, where cure is not possible. The COPD patients, they live long, if not always completely well with their COPD. Diabetes patients and heart patients, they have a longer life expectancy.*

(Hospital physician, Group 1)

Whereupon the physician in the municipal health service replied, ‘*But they may also enter a palliative phase with their illness, I think.*’ (GP, Group 1).

In connection with the palliative care team in the specialist health service, other diagnostic groups were reported to have received assistance from the team, but these appeared to be a few individual cases:

*In the palliative care team, there have been almost only cancer patients, but we have had the occasional advanced COPD patient who has been under the team.*

(Hospital physician, Group 2)

The transition from treatment focus to relief was also perceived differently by the physicians. Among the physicians in the municipal health service, the transition to palliative treatment was described as gradual and smooth. The physicians described how the patients’ situation and needs governed the transition:

*Palliative care often refers to the last stage of life, but it is the symptom pressure that determines the need.*

(GP, group 2)

To distinguish which patients had palliative needs, they pointed to experience, relationships over time and familiarity with the patients:

*When I know the patient well and we have spent time together, I feel that we are now in ‘that phase’. It is difficult to say exactly what determines it. It’s a feeling, an experience you make yourself.*

(GP, Group 1)

While there was a description of gradual transitions to palliative treatment among physicians in the municipal health service, the physicians in the specialist health service described a clearer and more criteria-based transition from curable to incurable disease and transition to the palliative phase:

*We delineate the palliative phase quite sharply; is the patient being treated for curative purposes, or is the aim palliative? The decisive thing is, is this a patient that we are doing everything to be able to heal or has the patient’s illness reached a stage where it is not possible?*

(Hospital physician, Group 2)

### 3.2. Theme 2: Alternating Understandings of Roles

Overall, the physicians had a collective understanding that relief is a physician’s core task because healing will not always be possible. However, it was also clear that the understanding of the role of the specialist healthcare service and the primary healthcare service differed. In this sense, the specialist provides active physical relief, whereas the generalist provides long-lasting comfort.

A hospital physician explained that the role was to relieve symptoms:

*After all, we are trying to make the symptoms less bothersome. Whether it’s nausea or pain. … My role mainly consists of making assessments … making decisions about which medicines to give and measures to take, discussions about such things.*

(Hospital physician, Group 2)

While the hospital physician said that he could attend to a patient off and on over time, either because the patient was hospitalised for a short period or attended appointments at the outpatient clinic, the GP believed their role was to be fully engaged with the patient’s care and remain involved until the end of palliative care.

*I am in the middle of the situation, the communication with the patient, communicating with the people around the patient … and in the assessments that must be made continuously during the course.*

(GP, Group 2)

Another GP added that their role was largely focused on acting as a consultant, especially for the nurses providing home care, and being in dialogue with all parties involved, determining responsibilities and knowing which specialists one should seek guidance from at all times.

While the hospital physicians were inclined to explain their activity as episodic and in clinical terms referring to, for example, chemotherapy, immunotherapy and names of drugs, the GPs and nursing home physicians tended to talk more about communication, problem-solving and interaction over the long term. They gave the impression of having a tacit agreement that their role focused on ensuring palliative patients stay in their own homes or nursing homes as long as possible. At the same time, it was clear that the hospital physicians played the leading role in the first phases of a palliative course for many patients. A hospital physician explained and exemplified cancer treatment:

*In an early palliative course, they often come to the cancer outpatient clinic and receive treatment in the form of chemotherapy, but eventually, when the tumour-directed treatment is finished … some patients want things arranged in order to die at home … which places great demands on the GP and the home care service … and to relatives of course. The impression is that GPs play a more central role at that stage than they do early in the process when the patient is healthy enough to get to the cancer clinic, and it is also a point that they come there to get the treatment they need.*

(Hospital physician, Group 2)

The GP followed up by saying:

*GPs can be more or less connected, but what is appropriate is different…. I think it is important not to have too strict an idea of what is right. We also have to consider what is the most effective level. Sometimes it can actually be most effective that it is the hospital physician who is closest to you. Sometimes it is perhaps the case that the patient does not want to have contact with the GP because the GP is considered to have been too late to make the diagnosis in the first place.*

(GP, Group 2)

Broad agreement was evident among the physicians that patients’ and relatives’ expectations of the physician’s role could be enormous and at times unrealistic. A nursing home physician believed that society, in general, is characterised by a perception that there is a cure for most things:

*We see this especially in relatives and patients who are younger, perhaps not the 80+ generation … so we spend a lot of time explaining that this cannot be treated. You won’t recover from this.*

(Nursing home physician, Group 1)

Aligning the expectations of both patients and relatives was thus an important task. But the physicians also had expectations of each other, depending on whether they worked in a hospital, nursing home or as a GP, although not always expressed explicitly. A nursing home physician believed that the focus on treatment had led to more uncertainty about what the goal was when patients were discharged from the hospital:

*The patient may have received intensive treatment in the hospital before coming to the nursing home, and then I am unsure of what is expected. Should the patient be readmitted if worsening? The hospital physician provides good information about what has been carried out but does not clarify what the plan is.*

(Nursing home physician, Group 1)

The hospital physician replied: *The goal is palliation. You can have very active treatment even if the patient is considered palliative.* (Hospital physician, Group 1).

One of the GPs had previously worked as a hospital physician and told of an experience with failed guidance to another GP:

*I was new and idealistic in the profession, but the GP got very annoyed with me and said, ‘I don’t like what you’re doing right now. The patient is on my list and is my responsibility’. As a hospital physician, I was a little surprised because I just wanted to be helpful. But he was absolutely right. The patient was his responsibility and he had known him for a long time. The GP was absolutely prepared to take his responsibility.*

(GP, Group 3)

The discussion between the physicians gave the impression that everyone was careful not to step into each other’s responsibilities and roles. However, at the same time, they described some unfortunate exchanges that could have been avoided with greater openness. A GP said:

*I have experienced over the years that palliative care in collaboration with hospital physicians or the palliative care team can be brilliant, but sometimes there is complete confusion of roles and chaos about who does what. A dialogue must be created so that we can instead supplement each other.*

(GP, Group 3)

### 3.3. Theme 3: Absence of Planning

What was perceived as a tendency towards unclear or implicit role expectations between the physicians also affected how they experienced the interaction regarding the palliative patients. The interaction was made difficult when they experienced a lack of planning or when other physicians did not follow up as expected. A nursing home physician was particularly frustrated that there were often no plans for patients after a hospital stay:

*Recently we had a patient who came from the hospital with intravenous broad-spectrum antibiotics. He came on a Friday evening, and on Saturday he died. Nothing was made clear to the patient or the relatives while he was admitted. He was reported as ‘palliative’ with a short life expectancy … this happens too often…. I don’t think it’s okay.*

(Nursing home physician, Group 3)

Such cases were not only difficult for the nursing home physician’s follow-up but were also perceived as unfortunate for the patient. The nursing home physician explained:

*Some patients say that they are sent to the nursing home with a pat on the back and are told to see how it goes … those who are cognitively healthy can say that, ‘I understood what they meant’, but they experienced that the staff at the hospital did not want to talk to them about the disease.*

(Nursing home physician, Group 3)

The formal lines of communication between the physicians were stated to be the epicrises sent after hospital treatment and electronic nursing and care reports, which were primarily written by and addressed to the nursing staff. The municipal physicians experienced the electronic care reports as particularly important because they were most often received earlier than the hospital physician’s epicrisis. The hospital physicians, for their part, believed that they gave a clear message to physicians in the municipality when the patient’s condition changed into a palliative course, at least in the case of cancer patients.

*It is basically communicated through epicrises, so it may be that if there is a need for supplementary information, it is of course possible for nursing home physicians or GPs to call to get further information or to discuss the patient, it does happen.*

(Hospital physician, Group 2)

A GP followed up by saying:

*Regarding communication challenges, I think the biggest mistake that can be made is that healthcare professionals talk too much to each other and not to the patient.*

(GP, Group 2)

All the physicians mentioned the importance of providing valuable information to patients and relatives, but some experienced challenges related to confidentiality and cases where the patient wanted to limit relatives’ access to information. While the municipal physicians were concerned about creating a good dialogue with relatives through collaboration with the patient, the hospital physicians gave the impression of having less contact with relatives. A hospital physician said:

*I may not have the impression that we, at the hospital, have that much contact with relatives. Although we are open, of course it happens that relatives call and want to talk to us.*

(Hospital physician, Group 2)

In general, the physicians were unclear about how they collaborated to facilitate good palliative care. The impression was equally clear that the need for treatment clarification and planning was great both among hospital physicians and municipal physicians. While the need for clarification was common, what they felt needed clarification seemed to vary. The hospital physicians seemed to emphasise the medical treatment and its effect, whereas the municipal physicians struggled more with the need to plan and clarify what quality of life meant for the patient. 

Hospital physician:

*I sometimes have the feeling that we do too much … and then it’s not good enough anyway, because we should have stopped earlier. It is not so easy when you stand there alone. It is good when it can be discussed in a collegium. And with the patient and relatives.*

(Hospital physician, Group 1)

GP:

*It is important to clarify what it is possible to achieve in terms of health for the person who is ill, and when treatment becomes more of a nuisance than a benefit. And then there are some more unpleasant clarifications, also in relation to how much others should be involved, what the patient really wants. There may be things that are a little more difficult to clarify, but which must take some time and which you have to deal with. The health service can contribute something, but that is not enough, you have to have a network and other supporters… The health service cannot extinguish all problems in a life. So, it is important to see our limitations as well.*

(GP, Group 2)

## 4. Discussion

The findings in this study show that, regardless of service level, the physicians had a collective understanding that their goal in palliative care was to contribute basic medical competence to relieve ailments and facilitate the best possible quality of life for patients with incurable diseases. A common opinion also prevailed that palliative care meant active treatment and was not reserved for the terminal phase of life. At the same time, the participants had nuanced perceptions of who the palliative patient was and what role and responsibility they, as physicians, had in the palliative process. Where the physicians in the municipalities had a more open attitude towards the palliative patients being individuals with chronic and long-term disorders, such as COPD and dementia, the hospital physicians were still mostly concentrated on the cancer patient as the typical receiver of palliative care. The hospital physicians were also more oriented towards physical symptom relief and short-term treatment efforts, whereas the physicians in the municipality believed that they were in long-term relationships with both patients and families and other health professionals. Moreover, municipal physicians often expressed more holistic thinking to achieve a greater quality of life. These findings are confirmed by several studies [10,35,36]. In their own way, the physicians at both levels of the service experienced unclear expectations of their roles and assignments, which could cause problematic transitions for the patients, as also confirmed by previous research [22]. Challenging transitions were characterised by little communication, few clarifications and an implied attitude that physicians at the cooperating service level had to make their own decisions with little support. 

Previous research has shown that GPs feel that they lose control over their patients when the specialists take over and that they may be reluctant to confront the hospital physicians due to fear of reprimands, mistrust, or lack of recognition [37]. The municipal physicians’ need to clarify what the hospital physicians expected was clear in our interviews, but it did not seem that they were in the habit of asking for such clarifications. The hospital physicians, for their part, could also perceive that municipal physicians as well as patients had unclear, often unrealistic expectations of what hospital treatment should achieve. What was striking in the interviews was that the expectations did not seem to be communicated or requested, but remained implicit and could lead to reproaches. While the GPs blamed the hospital physicians for not informing them about the goal for further treatment, the hospital physicians believed that the goal was obviously palliation and that they were always open to inquiries if anything was unclear. The hospital physicians stated this offer applied both to physicians in the municipality and the patients themselves or their next of kin. Moreover, the hospital physicians expressed that they refrained from setting conditions for patient treatment after discharge out of respect for the GP’s responsibility. 

Our interviews revealed that communication between the physicians often occurred in written form through transfer reports (epicrises), but there was a perceived need for more oral dialogue and opportunities for discussion and planning. Advance Care Planning (ACP) is recommended by authorities, but it is infrequently used in Norway, albeit showing increasing adoption [38]. Internationally, it is advised that GPs should initiate ACP [39], but they may encounter challenges when discussing end-of-life care with certain patients, especially when patients receive treatment in hospitals [15]. Furthermore, the identification of the “main provider for palliative care” appears unclear in the Norwegian context. Our findings indicate that individual physicians take responsibility for their respective levels of care, but there lacks a coordinated plan, such as Advance Care Planning (ACP), that can be collaboratively agreed upon and utilised as a guiding framework.

The organisation of the health service can be viewed as consisting of several social systems that are functionally differentiated [27]. Each system observes and relates to the environment based on its codes and semantics, which may conflict when they interact in the delivery of services [27,40]. Consequently, independent professional regulations and frameworks for patient treatment set by the level at which the physicians operate, characterise their interaction. However, codes can also be seen as frameworks for understanding, such as the understanding of what palliative care is and which patients should be provided with services. For example, the paired discussions revealed that the physicians did not have an agreed understanding of the transition to a palliative phase. While the hospital physicians pointed to clear and criteria-based transitions where the possibility of curing the disease was no longer present, the GPs perceived the transition as gradual and dependent on interpretations based on familiarity with the patient.

The social systems to which physicians belong are further affected by external conditions, such as laws and economics, where the codes are based on responsibility/duty and profitability and do not necessarily consider science or the patient’s quality of life. When several external codes affect service delivery at the same time, a polyphony [41] arises where the professionals have to choose what is most important in the given context. According to Vik and Hjelseth [41], interaction can be understood by looking at how professionals relate to the various expectations rather than the expectations themselves.

In our findings, although the prevailing expectations were sometimes implicit, the physicians at both levels expressed frustration in situations where the interacting physician did not act as expected. How the physicians dealt with expectations can be interpreted in our findings as a withdrawal. The physicians at both levels appeared to be waiting and passive when it came to planning the palliative course. Although the need to plan and clarify was present at both levels of the service, clear communication breakdowns were evident that reinforced the impression that the physicians concentrated on their own responsibilities and refrained from interfering in those of others.

The lack of clarity in the roles and expectations between the physicians and the problems that this can cause, was evident in our findings, as in many other studies [42,43,44]. But physicians also described evasions in communication with patients and their relatives. Examples were given of patients receiving limited information, which had to be read between the lines, or that information could be given to relatives, but preferably only if it was requested. A Spanish study [45] found that healthcare professionals, to spare patients and their families from stress and worry, avoid naming or openly discussing the purpose of palliative care. Such avoidance contributes to maintaining myths, misunderstandings and a lack of awareness about palliative care within society. Specifically, a lack of transparency about what palliative care is and who the target group is in itself a barrier to implementation worldwide [18,46].

Open discussions are needed about the polyphony in the codes that influence the professionals’ assessments and actions. Moreover, designated meeting spaces where interacting professionals can discuss what they expect and how they can fulfil such expectations are also needed. Physicians’ interaction between the primary and specialist healthcare services regarding palliative patients appears as a fragmented division of tasks rather than a real collaboration with joint responsibility, which is what the palliative patient group needs.

### Strengths and Limitations

The low number of participants in this study can be seen as a limitation and is possibly due to the recruitment taking place in a limited selection of municipalities (predetermined based on participation in a previous project) and the ongoing pandemic, which created increased workloads for physicians in both hospitals and municipalities. Previous studies also point to challenges in recruiting physicians for qualitative studies [47]. We attempted to address this challenge by extending the project period and making arrangements for online interviews. The physicians also received remuneration for lost earnings. 

Although the sample was small, we consider that employing pair interviewing is a major strength of the study and the dialogue between the physicians was experienced as committed and strong. The interview format made it possible for the perspectives to be shared in a here-and-now situation that enabled spontaneous responses and what we as interviewers perceived as open and honest statements from both parties. As the discussion took place between two or three participants, everyone was given plenty of time to offer and elaborate on their perspectives as well as consider those of their co-participants. 

The fact that both researchers are nurses can be seen as a strength concerning bias and the ability to ask exploratory questions. We experienced that the physicians primarily participated in a conversation among colleagues and did not adapt their communication because the researchers represented a different professional group. Among the physicians, both sexes were represented, and they had many years of experience in the profession and in treating palliative patients. However, we got the impression that the selection of hospital physicians represented expertise and responsibility particularly related to cancer diagnoses. The examples provided by the physicians predominantly pertained to cancer treatment. The explanation may be that the gatekeepers who assisted with the recruitment referred to physicians responsible for cancer patients, reflecting the traditional perception of which patients are seen as ‘palliative’.

Lastly, the context and the participants are described in as much detail as confidentiality allows and the findings are substantiated with quotes from both medical perspectives. The findings are discussed in the light of theory and previous research and the research process is communicated transparently so that the transfer value of the contribution can be assessed.

## 5. Conclusions

The findings in this study show that physicians’ interaction between the primary and specialist healthcare services concerning palliative patients appears as a fragmented distribution of tasks rather than a real collaboration with shared responsibility. The physicians at both levels appear to take withdrawn roles as a reaction to unclear and unspoken expectations and to avoid interfering with others’ responsibilities. Perceptions of palliative care and which patients should be included also varied. To realise full collaboration between physicians that benefits both patients and physicians, greater openness and real arenas for discussion and decision-making support are required. 

## Data Availability

Audio recordings have been deleted and anonymised transcripts have been stored in line with guidelines provided by the Norwegian Agency for Shared Services in Education and Research and Nord University.
